# Knowledge, Attitudes, and Practices With Regard to Cosmetic Procedures Among the General Population in the Western Region of Saudi Arabia: A Cross-Sectional Study

**DOI:** 10.7759/cureus.52214

**Published:** 2024-01-13

**Authors:** Mohammed F Bondagji, Eyad E Sindi, Ghadeer E Alamri, Sarah M Fageeh, Ahmed A Niyazi, Dhuha O Alquhra Alotaibi, Ghadi A Alghamdi, Adel A Alluhaybi, Mokhtar Shatla

**Affiliations:** 1 Medicine and Surgery, College of Medicine, Umm Al-Qura University (UQU), Makkah, SAU; 2 Family Medicine, College of Medicine, Umm Al-Qura University (UQU), Makkah, SAU

**Keywords:** saudi arabia, practice, attitudes, knowledge, cosmetic procedures

## Abstract

Background

One of the most frequently carried out medical procedures is an aesthetic procedure. These procedures have become increasingly popular in our country due to various factors, such as body image dissatisfaction, the desire for perfection, and the expanding influence of social media. The aim of our study is to assess the knowledge, attitudes, and practices with regard to cosmetic procedures among the general population in the western region of Saudi Arabia.

Methods

This population-based cross-sectional study uses a self-report questionnaire distributed via social media platforms with randomized sampling between March and April 2023. The target population of this study is the general population in the western region of Saudi Arabia who are 18 years old and above from both genders. All participants who have congenital anomalies or mental disorders, are non-Arabic speakers, and refuse to participate in the study are excluded. Our data collected via an online self-administrated translated questionnaire survey was designed using a Google Forms questionnaire template.

Results

The study questionnaire was completed by 477 individuals in total. The mean age of the participants was 27.4±12.9 years, with ages ranging from 18 to over 60. A total of 190 individuals (39.8%) had prior knowledge of cosmetic procedures, and 338 (70.9%) were female. Of the non-surgical cosmetic treatments that people are most familiar with, 37.9% mentioned filler injections. Additionally, the most popular surgical cosmetic surgery was rhinoplasty (73.6%) and liposuction (22%). 85.7% of respondents said social media impacts people's decisions to undergo cosmetic treatments, while 86.8% of respondents said women do more cosmetic procedures than males. Of the participants, only 40 (8.4%) had previous cosmetic procedure done before. Rhinoplasty was the most common undergone surgical treatment (15.4%). In terms of non-surgical treatments, hair removal accounted for the majority (51.3%).

Conclusion

This study revealed that 39.4% of the study population, the majority of which are young individuals, females, and singles, have a good knowledge with regard to cosmetic procedures due to many factors such as culture diversity among generations, distribution of the social media, and interest differences. We conclude that the social media factor can significantly influence the practice of cosmetic procedures.

## Introduction

One of the most frequently performed medical procedures is aesthetic surgery. These procedures have gained increasing popularity in our country due to various factors, including dissatisfaction with one's body image, the pursuit of perfection, and the growing influence of social media [1]. Cosmetic procedures are defined as "treatments designed to enhance a person's appearance" [2] and are categorized into surgical and non-surgical options [3]. Surgical procedures encompass rhinoplasty, breast augmentation, and blepharoplasty [4], while non-surgical procedures include botulinum toxin injections, chemical peels, filler treatments, vein removal, laser skin resurfacing, and laser hair removal [5,6].

According to the American Society of Plastic Surgeons, there has been a significant global increase in the number of cosmetic procedures performed over the past two decades, with a remarkable growth rate of 169% between 2000 and 2019. Notably, the majority of these procedures were minimally invasive, and women have shown a greater willingness to undergo these minimally invasive procedures, as they offer a more natural appearance with minimal recovery time [7].

Several epidemiological variables, social factors, and psychological traits, including self-esteem, body image, and other personality characteristics, as well as specific psychopathologies have been associated with an interest in cosmetic surgery. These variables can predict an individual's inclination to pursue or avoid cosmetic procedures [8].

It is worth noting that Saudi Arabia ranks 22nd among the top 25 nations in the world with the highest rates of cosmetic surgery, according to a study conducted by the International Society of Aesthetic Plastic Surgery [9]. Numerous studies have also explored surgical and non-surgical procedures in various cities and regions across Saudi Arabia. However, there has been a notable absence of prior research specifically addressing the western region of the country. Hence, our study aims to evaluate the knowledge, attitudes, and practices with regard to cosmetic procedures among the general population residing in the western region of Saudi Arabia.

## Materials and methods

This is a population-based cross-sectional study conducted at Umm Al-Qura University, Makkah, Saudi Arabia, from March to April 2023, employing a self-report questionnaire distributed via social media platforms with randomized sampling. The study's target population comprises adults aged 18 and above from the western region, including both genders. Individuals with congenital anomalies and mental disorders, who are non-Arabic speakers, or who decline to participate are excluded from the study. The questionnaire comprises five sections: a consent form, sociodemographic data, an assessment of knowledge regarding cosmetic procedures, an evaluation of attitudes toward cosmetic procedures, and an assessment of the practice of cosmetic procedures. The selection of items for the questionnaire was informed by a thorough literature review [10]. The questionnaire was distributed in Arabic language. Our data were collected through an online, self-administered, translated questionnaire survey created using a Google Forms questionnaire template. It was accompanied by information about the survey's objectives and the target population and a request for voluntary participation, with the assurance of maintaining the confidentiality of all responses.

Sample size determination

The minimum required sample size for this study was determined using OpenEpi Version 3.0, taking into account the following factors: a population size estimated at approximately 8,325,304 individuals (as reported by the General Authority for Statistics), a confidence interval (CI) level of 95%, and an anticipated frequency of 50%. The calculated sample size came out to be 385 participants. To account for potential data loss, the total sample size was increased to 400 participants. This study received approval from the Biomedical Research Ethics Committee of Umm Al-Qura University, with the approval number HAPO-02-K-012-2023-03-1519.

Data analysis

The data were collected, reviewed, and subsequently input into IBM SPSS Statistics for Windows, Version 21.0 (Released 2012; IBM Corp., Armonk, New York, United States). All statistical methods employed were two-tailed, with a significance level (alpha) set at 0.05, considering results as significant if the p-value was less than or equal to 0.05. Descriptive analysis involved the presentation of frequency distributions and percentages for various study variables, including participants' personal information and cosmetic procedures undergone personally and within their families. Additionally, data pertaining to knowledge, attitudes, and practices with regard to cosmetic procedures were tabulated.

Cross-tabulation was utilized to display factors associated with participants' knowledge of cosmetic procedures, and statistical significance was assessed using the Pearson chi-squared test. In cases where small frequency distributions were observed, an exact probability test was applied.

## Results

A total of 477 eligible participants completed the study questionnaire. Participants' ages ranged from 18 to more than 60 years with a mean age of 27.4±12.9 years old. Exactly 338 (70.9%) were females, and 342 (71.7%) were single while 118 (24.7%) were married. Exactly 455 (95.4%) were Saudi Arabians, of which 290 (60.8%) had diploma/bachelor's degree. A total of 298 (62.5%) were students, and 90 (18.9%) were employed. A monthly income of less than 5000 Saudi riyal (SR) was reported among 304 (63.7%) participants, while 64 (13.4%) had a monthly income of 5000-9000 SR and 77 (16.1%) 10000-19000 SR (Table 1).

**Table 1:** Sociodemographic data of study participants in the western region of Saudi Arabia SR: Saudi riyal

Sociodemographic data	Number	%
Age in years		
18-29	354	74.2%
30-39	38	8%
40-49	42	8.8%
50-59	29	6.1%
60+	14	2.9%
Gender		
Male	139	29.1%
Female	338	70.9%
Marital status		
Single	342	71.7%
Married	118	24.7%
Divorced/widowed	17	3.6%
Nationality		
Saudi	455	95.4%
Non-Saudi	22	4.6%
Educational level		
Secondary/below	172	36.1%
Diploma/bachelor	290	60.8%
Post-graduate	15	3.1%
Employment		
Unemployed/retired	74	15.5%
Student	298	62.5%
Employed	90	18.9%
Free works	15	3.1%
Monthly income		
<5000 SR	304	63.7%
5000-9000 SR	64	13.4%
10000-19000 SR	77	16.1%
20000-30000 SR	18	3.8%
>30000 SR	14	2.9%

As for participants' awareness and perception regarding cosmetic procedures, the current study showed that 190 (39.8%) had previous knowledge about cosmetic procedures. With regard to the most common non-surgical cosmetic procedures they were aware of, 181 participants (37.9%) spoke of filler injection, 145 (30.4%) reported for hair removal, and 85 (17.8%) told about botulinum toxin A. In addition, when it came to surgical cosmetic procedures, rhinoplasty and liposuction were the most well-known surgical cosmetic procedures, with 351 (73.6%) of participants recognizing rhinoplasty, followed by liposuction 105 (22%) and breast augmentation 22 (4.4%). As for reason that motivates people to undergo cosmetic procedures, 324 (67.9%) cited the influence of social media, and 319 (66.9%) mentioned a desire for better appearance. Additionally, 199 (41.7%) mentioned other factors, and 173 (36.3%) reported for personal desire. The most reported sources of information included the internet 366 (76.7%), friends 160 (33.5%), and physicians 145 (30.4%) (Table 2).

**Table 2:** Participants' awareness and perception regarding cosmetic procedures in the western region of Saudi Arabia

Cosmetic procedure awareness	Number	%
Do you have previous knowledge about cosmetic procedures?		
Yes	190	39.8%
Maybe	139	29.1%
No	148	31%
The most common non-surgical cosmetic procedures		
Filler injection	181	37.9%
Hair removal	145	30.4%
Botox	85	17.8%
Laser skin regeneration	34	7.1%
Non-surgical fat reduction	32	6.7%
The most common surgical cosmetic procedures		
Liposuction	105	22%
Rhinoplasty	351	73.6%
Breast augmentation	21	4.4%
The most common reason that motivates people to undergo cosmetic procedures		
Social media effect	324	67.9%
To have a better look	319	66.9%
Surrounding people effect	199	41.7%
Personal desire	173	36.3%
Fashion	142	29.8%
Others	17	3.6%
Source of information for cosmetic procedures		
Internet	366	76.7%
Friends	160	33.5%
Physicians	145	30.4%
TV	87	18.2%
Others	86	18%

The study declared that about 418 (87.6%) of the participants think cosmetic procedures are common, 414 (86.8%) stated that women undergo more cosmetic procedures than men, 409 (85.7%) believe that social media influences the decision to have a cosmetic procedure, and 373 (78.2%) agreed that low self-esteem causes people to undergo cosmetic procedures, while 354 (74.2%) indicated that they would feel intimidated if they decided to do cosmetic procedures (Table 3).

**Table 3:** Participants' attitude and perception regarding cosmetic procedures in the western region of Saudi Arabia

Attitude items	Yes		No		Don't know	
	No	%	No	%	No	%
Agree to cosmetic procedures	158	33.1%	229	48%	90	18.9%
You think cosmetic procedures are common	418	87.6%	37	7.8%	22	4.6%
You believe that cosmetic procedures are acceptable in society	219	45.9%	125	26.2%	133	27.9%
You consider cosmetic procedures a waste of money	282	59.1%	115	24.1%	80	16.8%
People who have undergone cosmetic procedures look better and more attractive	134	28.1%	211	44.2%	132	27.7%
If you decide to have any cosmetic procedure, can you tell others?	279	58.5%	121	25.4%	77	16.1%
If you decide to do cosmetic procedures, will you feel intimidated?	354	74.2%	66	13.8%	57	11.9%
You will undergo cosmetic procedures at the request of others	23	4.8%	436	91.4%	18	3.8%
You believe that social media influences the decision to have a cosmetic procedure	409	85.7%	46	9.6%	22	4.6%
Low self-esteem causes people to undergo cosmetic procedures	373	78.2%	61	12.8%	43	9%
Women do more cosmetic procedures than men	414	86.8%	28	5.9%	35	7.3%

Only 40 (8.4%) participants previously undergone cosmetic procedures. The most undergone surgical procedures included rhinoplasty 6 (15.4%), breast augmentation 2 (5.1%), tummy tuck 2 (5.1%), and eyelid lift 2 (5.1%), while 20 (51.3%) had not undergone any surgical procedures. In terms of non-surgical procedures, the most commonly undergone were hair removal 20 (51.3%), filler injections 12 (30.8%), and Botox 13 (33.3%). Among those who had not undergone cosmetic procedures, the most reported reasons were self-satisfaction 322 (73.5%), religious concerns 191 (43.6%), and fear of complications 63 (14.4%) (Table 4).

**Table 4:** Self-practice regarding cosmetic procedures among study participants in the western region of Saudi Arabia

Self-practice	Number	%
Have you ever done cosmetic procedures?		
Yes	40	8.4%
No	437	91.6%
Type of undergone surgical procedure (n=40)		
Rhinoplasty	6	15.4%
Breast augmentation	2	5.1%
Tummy tuck	2	5.1%
Brow lift	2	5.1%
Others	7	17.9%
None	20	51.3%
Type of undergone non-surgical procedure (n=40)		
Hair removal	20	51.3%
Filler injection	12	30.8%
Botox	13	33.3%
Others	5	12.8%
None	8	20.5%
What is the reason for not doing cosmetic procedures?		
Self-satisfaction	322	73.5%
Religious concerns	191	43.6%
Fear of complications	63	14.4%
Financial concerns	53	12.1%
Others	70	16%

A total of 208 (43.6%) reported that one of their relatives had undergone cosmetic procedure. Among these, 53 (25.5%) mentioned siblings, 22 (10.6%) mentioned parents, and the majority 125 (60.1%) mentioned other relatives. The most commonly undergone non-surgical procedures among relatives were filler injections 120 (57.7%), Botox 97 (46.6%), hair removal 72 (34.6%), and non-surgical liposuction 51 (24.5%). As for surgical procedures, rhinoplasty 62 (29.8%) and liposuction 41 (19.7%) were the most commonly mentioned, while other procedures accounted for 54 (26%) (Table 5).

**Table 5:** Family history of undergoing cosmetic procedures in the western region of Saudi Arabia

Family history	Number	%
Has any of your relatives had cosmetic procedures?		
Yes	208	43.6%
No	269	56.4%
Type of relation		
Parents	22	10.6%
Siblings	53	25.5%
Partner	8	3.8%
Others	125	60.1%
Type of non-surgical procedure		
Filler injection	120	57.7%
Botox	97	46.6%
Hair removal	72	34.6%
Non-surgical liposuction	51	24.5%
Laser skin regeneration	16	7.7%
None	18	8.7%
Type of surgical procedure		
Rhinoplasty	62	29.8%
Others	54	26%
Liposuction	41	19.7%
Tummy tuck	31	14.9%
Breast augmentation	18	8.7%
None	70	33.7%

Among the participants, 156 (44.1%) of the younger age group were knowledgeable about cosmetic procedures, while only six (20.7%) of those aged 50-59 years were aware with recorded statistical significance (p=0.007). Moreover, 153 (45.3%) of females knew about these procedures, in contrast to 37 (26.6%) of males (p=0.001). Exactly 149 (43.6%) of the single individuals were familiar with cosmetic procedures compared to five (29.4%) from the divorced group (p=0.034) (Table 6).

**Table 6:** Factors associated with participants' knowledge about cosmetic procedures SR: Saudi riyal; P: Pearson X2 test; $: exact probability test; *: p<0.05 (significant)

Factors	Do you have previous knowledge about cosmetic procedures?						p-value
	Yes		Maybe		No		
	Number	%	Number	%	Number	%	
Age in years							0.007*
18-29	156	44.1%	107	30.2%	91	25.7%	
30-39	11	28.9%	9	23.7%	18	47.4%	
40-49	12	28.6%	10	23.8%	20	47.6%	
50-59	6	20.7%	10	34.5%	13	44.8%	
60+	5	35.7%	3	21.4%	6	42.9%	
Gender							0.001*
Male	37	26.6%	45	32.4%	57	41%	
Female	153	45.3%	94	27.8%	91	26.9%	
Marital status							0.034*
Single	149	43.6%	100	29.2%	93	27.2%	
Married	36	30.5%	33	28%	49	41.5%	
Divorced/widowed	5	29.4%	6	35.3%	6	35.3%	
Nationality							0.589
Saudi	179	39.3%	134	29.5%	142	31.2%	
Non-Saudi	11	50%	5	22.7%	6	27.3%	
Educational level							0.140^$^
Secondary/below	56	32.6%	54	31.4%	62	36%	
Diploma/bachelor	126	43.4%	81	27.9%	83	28.6%	
Post-graduate	8	53.3%	4	26.7%	3	20%	
Employment							0.146
Unemployed/retired	21	28.4%	23	31.1%	30	40.5%	
Student	132	44.3%	86	28.9%	80	26.8%	
Employed	32	35.6%	25	27.8%	33	36.7%	
Free works	5	33.3%	5	33.3%	5	33.3%	
Monthly income							0.332
<5000 SR	124	40.8%	93	30.6%	87	28.6%	
5000-10000 SR	26	40.6%	17	26.6%	21	32.8%	
10000-19000 SR	24	31.2%	21	27.3%	32	41.6%	
20000-30000 SR	7	38.9%	6	33.3%	5	27.8%	
>30000 SR	9	64.3%	2	14.3%	3	21.4%	

## Discussion

To the best of our knowledge, this study represents the first attempt to assess the knowledge, attitudes, and practices with regard to cosmetic procedures among the general population in the western region of Saudi Arabia. We discovered that 39.6% of the participants display a solid understanding of aesthetic procedures, a significantly higher proportion compared to a previous local study conducted in Al-Majmaah in 2020, which reported that only 18% possessed a good knowledge base [10]. Globally, a different study conducted in 2016 among undergraduates studying basic sciences at a Nigerian university found that only 4.2% of the respondents had a reasonably good level of knowledge [11]. This variance can be attributed to various factors, including cultural diversity and the widespread use of social media within our local society.

Among the three categories examined, namely, young individuals, females, and single participants, we observed the highest perception of cosmetic procedures. This outcome aligns with expectations, given the heightened sociocultural pressures exerted on women to conform to certain standards of physical appearance. Such pressures may explain the observed gender disparity. Similarly, previous studies conducted locally in Riyadh [12] and globally by Markey and Markey and Swami et al. [13,14] also found that women are more likely to embrace cosmetic surgery.

In terms of the acceptance of aesthetic procedures, only 158 (33.1%) of the survey participants expressed approval, a notably lower figure when compared to two studies conducted in other regions of Saudi Arabia [10,12]. This outcome aligns with expectations, considering that our western region is known for its conservative societal norms. Interestingly, 418 (87.7%) of the 477 participants acknowledged that cosmetic procedures are indeed prevalent in our society.

Among our participants, only 40 (8.4%) had undergone prior aesthetic procedures. In contrast, in the United States, the rate of cosmetic surgery among women stands at 24.1% [15], whereas a substantial 55.4% of Saudi women have undergone such procedures [16]. This significant disparity can be attributed to the preference among American women for invasive treatments like liposuction and breast augmentation [15].

Another notable finding in our study is that the majority of respondents use the internet as their primary source of information about cosmetics (76.7%). This discovery aligns with a local study conducted in Al-Taif City among female university students, which reported that the internet was the most prominent source of information for cosmetics (79.1%) [17]. Furthermore, most of our participants believe that social media exerts the greatest influence on their decision to undergo cosmetic procedures. Surprisingly, these findings diverge from a study conducted in the United Kingdom that examined factors influencing the likelihood of undergoing cosmetic procedures. This UK study found that individuals with lower self-ratings of attractiveness were more likely to opt for cosmetic surgery [18].

Strengths and limitations

This research represents the first conducted study in the western region of Saudi Arabia aimed at assessing the knowledge, attitudes, and practices with regard to cosmetic procedures among the general population. The sample encompassed a diverse range of age groups, genders, and social networks.

The first notable limitation of this study is its reliance on a cross-sectional survey design and self-reported data, which may not fully capture the dynamics of cosmetic trends accurately. Additionally, the study's sample was confined to individuals encountered within the western region, limiting the generalizability of the findings to the entire Saudi Arabian population. We recommend conducting further research in other regions of Saudi Arabia among the general population, as cultural and traditional differences may yield varying results.

## Conclusions

Our findings revealed that 39.4% of the study population, the majority of which are young individuals, females, and singles, have a good knowledge with regard to cosmetic procedures due to many factors such as culture diversity among generations, distribution of the social media, and interest differences. There was a significant association between the young age group individuals and increased knowledge and awareness of cosmetic procedures; the rise in awareness in young people is most likely due to social media usage as it is the most reported source of information in our study. We conclude that the social media factor can significantly influence the practice of cosmetic procedures.
